# Sleep state of the elderly population in Korea: Nationwide cross-sectional population-based study

**DOI:** 10.3389/fneur.2022.1095404

**Published:** 2023-01-09

**Authors:** Heewon Hwang, Kyung Min Kim, Chang-Ho Yun, Kwang Ik Yang, Min Kyung Chu, Won-Joo Kim

**Affiliations:** ^1^Department of Neurology, Wonju Severance Christian Hospital, Yonsei University Wonju College of Medicine, Wonju, South Korea; ^2^Department of Neurology, Severance Hospital, Yonsei University College of Medicine, Seoul, South Korea; ^3^Department of Neurology, Bundang Clinical Neuroscience Institute, Seoul National University Bundang Hospital, Seongnam, South Korea; ^4^Sleep Disorders Center, Department of Neurology, Soonchunhyang University College of Medicine, Cheonan Hospital, Cheonan, South Korea; ^5^Department of Neurology, Gangnam Severance Hospital, Yonsei University College of Medicine, Seoul, South Korea

**Keywords:** sleep, aging, sleep qualities, daytime sleepiness, insomnia

## Abstract

**Objective:**

Interest in sleep disorders among the elderly, especially those in Korea, has increased. We aimed to describe the overall sleep status of the elderly population in Korea using survey data and to determine the risk factors concerning different aspects of sleep status.

**Methods:**

We conducted a cross-sectional survey on 271 respondents aged 65–86 years old. We performed multistage clustered random sampling according to the population and socioeconomic distribution of all Korean territories. The survey questionnaire was used to perform a structural assessment of sociodemographic characteristics; medical comorbidities; psychiatric comorbidities; and sleep status, including sleep duration, sleep quality, presence of insomnia, excessive daytime sleepiness, sleep apnea, and restless legs syndrome.

**Results:**

Approximately 12.5, 22.%, and 51.3% of the elderly population had poor sleep quality, excessive daytime sleepiness, and insomnia, respectively. Hypertension, dyslipidemia, insomnia, anxiety, and depression predicted poor sleep quality. Female sex, insomnia, and sleep apnea predicted excessive daytime sleepiness. Poor sleep quality and depression predicted insomnia.

**Conclusion:**

A substantial proportion of the elderly Korean population have sleep problems, including poor sleep quality, excessive daytime sleepiness, and insomnia. Sleep status is influenced by various factors, including age, sex, and metabolic and psychiatric comorbidities.

## 1. Introduction

South Korea has the fastest-aging population in the world. According to the Korean Statistical Information Service, individuals aged > 65 years comprised only 7.2% of the population in 2000, but >10.2% in 2008 and is expected to exceed 20% by 2025 (1). Despite the growing demand for medical care for the elderly population, medical infrastructure and personnel remain insufficient (2). With increasing numbers of the elderly, an increase in sleep disorders in the elderly population has been noted; however, sleep disorders have not been studied to the extent that chronic neurodegenerative or metabolic diseases have. Among the elderly, poor sleep quality causes deterioration of basic cognitive function, memory, and performance (3, 4). Furthermore, insomnia and inconsistent sleep duration are associated with metabolic syndrome (5, 6). In a recent study of the elderly population aged 65 years or older, there was also a study that self-reported poor sleep quality and self-reported too short or too long sleep duration were associated with an increased risk of falls (7). Although not targeting only the elderly, a nation-wide cohort study in Denmark showed that sleep disorder was associated with a high suicidal rate even after adjusting for other psychiatric comorbidities (8).

The duration of lighter sleep stages increases with aging, with more frequent episodes of nocturnal waking up. Moreover, the total sleep time decreases with age (9). Furthermore, the quality of sleep worsens with aging in combination with anxiety, chronic illness, and lack of social support (10). In addition to these aging-related physiological changes, sleep problems are strongly influenced by sociological factors (11). Sociologic factors affecting sleep quality have been studied in several countries (12–15). Poor sleep quality is prevalent among the rural elderly population (16, 17). Accordingly, in addition to drug and cognitive behavioral therapy, modulating sociodemographic factors in the elderly population is crucial to address sleep disorders (10, 18). Determining the sleep status of elderly individuals is important in choosing an appropriate intervention (19).

Recently, studies have emerged that COVID-19 infection affects sleep. In one study conducted in a group of 300 patients aged 18 years or older, poor sleep quality was observed in 64.8% of the follow-up results 6 months after confirmation (20). In addition, in the group of 1,733 discharged patients followed for about 6 months after diagnosis of COVID-19, the most common symptom was fatigue or muscle weakness followed by sleep difficulties (21). In a French study in which 1,736 participants responded, the incidence of posttraumatic stress disorder after an unprecedented lockdown was 17.5%, and it was found that anxiety, depression, and sleep problems developed high in this group 1 month later (22). Furthermore, there is also a study that psychological distress and anxiety have a high incidence rate in the COVID-19 patient group, and insomnia is a risk factor (23).

Based on these studies, we cannot rule out the possibility that the prevalence of psychiatric and general health comorbidities may have changed in demographic data before and after COVID-19 (20, 24, 25). This pandemic is thought to adversely affect the sleep behavior of the elderly population and increase risks such as falls, but studies on the relationship between the COVID-19 pandemic and sleep in the elderly are lacking (26). However, the data used in this study were collected before 2019, so COVID-19-related content was not included.

Nationwide cross-sectional studies on sleep are being conducted in many countries, and various conclusions have been drawn from each study. A study in China estimated that one in 10 of the population studied had sleep disorders (27). In addition, this study confirmed that sleep quality was correlated with sociodemographic data such as sex, marital status, and education level. According to another recent nationwide cross-sectional study conducted in China, excessive sleep duration rather adversely affects the cognitive function of the elderly population (28). In addition, a US study of 25,962 people with an average age of 48.1 years concluded that sleep duration was independently associated with a high incidence of depression (29). A nationwide cross-sectional study in Indonesia showed that aging is a risk factor for poor sleep quality, which can lead to depression, and stressed that managing such sleep quality is important to lower the incidence of depression in communities (30). As such, since sleep status is closely related to sociodemographic data, studies according to the characteristics of the population in each region are needed.

However, research on the sleep status of the elderly population in Korea is still lacking (31–34). Accordingly, determining the predictive factors for sleep quality, daytime sleepiness, and insomnia could facilitate the integrated management of elderly patients. We aimed to examine the overall sleep status of the elderly population in Korea and the various predictive factors for sleep disorders.

## 2. Materials and methods

### 2.1. Setting

We analyzed data from a nationwide cross-sectional survey of headache and sleep of Korean adult population aged 65–86 years, excluding Jeju-do.

South Korea was geographically divided into 16 administrative division at the time of study, and each administrative division was further divided into basic administrative units (si, gun, or gu). We performed multistage clustered random sampling according to the population and socioeconomic distribution in all Korean territories, except Jeju-do. First, 15 administrative divisions of South Korea (do), except Jeju-do, were designated as primary sampling units, and appropriate sample numbers were assigned to each primary sampling unit according to population distribution. In the second stage, we selected representative basic administrative units (si, gun, and gu) for each primary sampling units, and assigned a target sample number (35). Total 311 respondents were included.

The respondents gave their informed consent for face-to-face interviews using questionnaires regarding sleep status conducted by experienced interviewers hired by Gallup Korea. To minimize potential interest bias, the interviewers were informed that the survey was on a social health issue rather than a sleep issue.

The survey questionnaire was used to perform a structural assessment of sociodemographic characteristics including educational attainment, occupational status, drinking behavior, presence of lack of exercise, obesity, shift work, and taking sleep pill; cardiovascular and metabolic comorbidities, including hypertension, diabetes mellitus, dyslipidemia, and cardiovascular diseases; and psychiatric comorbidities, including depression and anxiety.

### 2.2. Definitions of sociodemographic characteristics

Educational attainment was divided into middle school graduation or less and high school graduation or higher. Occupation was considered to be present if the person answered the question asking what the job was. Alcohol consumption was considered to be a risk factor if alcohol was consumed on four or more days per week. Lack of exercise was considered as no exercise day during the week. Obesity was considered when the respondent had a BMI of 25 or higher based on the height and weight written by the respondent. In the questionnaire, respondents were asked if they worked shifts, and if they answered yes, they were defined as shift workers. In addition, it was defined as having a factor in taking sleeping pills if the respondents answered that they took sleeping pills at least once a week through the questionnaire. The presence of hypertension, diabetes, hyperlipidemia, and cardiovascular disease were all based on the respondents' description. Cardiovascular disease referred to coronary heart disease, stroke, peripheral arterial disease, and aortic disease.

### 2.3. Definitions of poor sleep quality, excessive daytime sleepiness, and insomnia

Sleep quality was assessed using the Pittsburgh Sleep Quality Index (PSQI) (36), which is a self-reported scale comprising seven components (subjective sleep quality, sleep latency, sleep duration, sleep efficiency, sleep disturbances, use of sleep medication, and daytime dysfunction). Based on the Korean version of the PSQI, poor sleep quality was indicated by a PSQI score ≥ 9 (37).

The Epworth Sleepiness Scale (ESS) was used to evaluate daytime sleepiness. Excessive daytime sleepiness (EDS) was indicated by an ESS score ≥ 10 (38).

The presence of insomnia was evaluated using the Insomnia Severity Index (ISI), which comprises seven items for diagnosing insomnia scored on a 0–4 scale. Subthreshold insomnia was indicated by an ISI score ≥ 8 (39).

### 2.4. Evaluation of depression and anxiety

Korean version of depression screening tool (Patient Health Questionnaire-9, PHQ-9) was used as screening tool. The Korean PHQ-9 was known as having 81.1% sensitivity and 89.9% specificity (40). In this study, depression was considered probable if the PHQ-9 score was five or higher (41). Goldberg Anxiety Scale (GAS) questionnaire was used to diagnose anxiety among participants. Participants were regarded as having anxiety according to GAS score ≥ 5, with a sensitivity of 82.0% and specificity of 94.4% (42).

### 2.5. Evaluation of sleep apnea and restless leg syndrome

The risk of sleep apnea was assessed using the Berlin Questionnaire (BQ), with a positive score in more than two or more categories indicating a high risk of sleep apnea (43). The presence of restless leg syndrome was assessed using the Cambridge-Hopkins Diagnostic Questionnaire; specifically, we used four essential diagnostic criteria proposed by the International Restless Legs Syndrome Study Group (44). If all four criteria were satisfied, the participant was considered to have restless leg syndrome. This was diagnosed by a neurologist based on a questionnaire.

### 2.6. Ethics

The study protocol was approved by the Institutional Review Board/Ethics Committee of Hallym University Sacred Heart Hospital in Korea (Approval no. 2011-I077). Written informed consent was obtained before the survey.

### 2.7. Statistical analysis

The normality of data distribution was assessed using the Kolmogorov–Smirnov test. After confirming normal data distribution, continuous variables were compared using Student's *t*-test, with an estimation of statistical significance under homogeneity of variances. The chi-square test was used to compare categorical variables. Finally, we performed binary multivariate logistic regression analysis to calculate the odds ratios (ORs) with 95% confidence intervals (CIs) for the occurrence of poor sleep quality, EDS, and insomnia. Multicollinearity between variants was minimized using the backward elimination method. Statistical significance was set at *p* < 0.05. Statistical analyses were performed using the Statistical Package for Social Sciences (version 26.0; IBM Corp., Armonk, NY, USA). This study was reported according to the STROBE (Strengthening the Reporting of Observational Studies in Epidemiology) checklist (45).

## 3. Results

### 3.1. Demographic characteristics

Among 311 initial participants aged > 65 years, 271 participants [age: 69.9 ± 4.4 years; 139 (51.3%) women] completed the survey. 139 (51.3%) participants were female. A total of 200 (73.8%) participants had an educational level lower than middle school. Moreover, 74 (27.3%) and 17 (6.3%) participants were unemployed and shift workers, respectively. Further, 142 (52.4%), and 72 (26.6%) participants did not exercise at all and were overweight or obese (body mass index > 25), respectively. Only 12 (4.4%) participants reported taking sleeping pills. Additionally, 123 (45.4%), 48 (17.7%), 86 (31.7%), and 15 (5.5%) participants had hypertension, diabetes mellitus, cardiovascular disease, and dyslipidemia, respectively (Table 1).

**Table 1:** Sociodemographic characteristics of the study population.

**Characteristics**	**Value (*N* = 271)**
Mean age (years)	69.9 ± 4.4
Female	139 (51.3)
**Level of education**	
Under middle school graduate	200 (73.8)
Over high school graduate	71 (26.2)
Inoccupation	74 (27.3)
Alcohol	67 (24.7)
Current smoker	39 (14.4)
Lack of exercise	142 (52.4)
Obesity	72 (26.6)
Hypertension	123 (45.4)
Diabetes mellitus	48 (17.7)
Cardiovascular disease	86 (31.7)
Dyslipidemia	15 (5.5)
Shift work	17 (6.3)
On sleeping pills	12 (4.4)
Sleep duration during weekdays (hours)	7.14 ± 1.38
Sleep duration during weekends (hours)	7.28 ± 1.39
Poor sleep health	34 (12.5)
Insomnia	139 (51.3)
Daytime sleepiness	62 (22.9)
Sleep apnea	53 (19.6)
Restless leg syndrome	28 (10.3)
Anxiety	45 (16.6)
Depression	64 (23.6)

Data are expressed as mean ± standard deviation or number (percentage).

Alcohol, refers to drinking more than four times a week; lack of exercise, refers to no exercise at all; obesity, refers to BMI ≥ 25; cardiovascular disease, refers to coronary heart disease, stroke, peripheral arterial disease, aortic disease; poor sleep quality, PSQI score ≥ 9; insomnia, ISI score ≥ 8; daytime sleepiness, ESS score ≥ 10; sleep apnea, BQ score ≥ 2; RLS, meets all four criteria of the CH-RLSq; anxiety, GAS score ≥ 5; depression, PHQ-9 score ≥ 5.

### 3.2. Sleep status

The average sleep duration during weekdays and the weekend were 7.14 ± 1.38 h and 7.28 ± 1.39 h, respectively. Thirty-four (12.5%) participants had poor sleep quality, based on PSQI scores ≥ 9; 139 (51.3%), 62 (22.9%), 53 (19.6%), 28 (10.3%), 45 (16.6%), and 64 (23.6 %) participants had insomnia, EDS, a high risk of sleep apnea, restless leg syndrome, anxiety, and depression, respectively (Table 1).

Furthermore, we compared sleep-related indicators according to gender. There was no difference in sleep duration according to gender, but PSQI, ISI, and ESS scores were statistically significantly higher in females (5.63 ± 2.71 vs. 4.80 ± 2.83, 10.90 ± 6.75 vs. 8.95 ± 6.69, 6.91 ± 4.30 vs. 5.71 ± 4.15, retrospectively) (Supplementary Table 1).

### 3.3. Subgroup analysis according to sleep quality, daytime sleepiness, and insomnia

#### 3.3.1. Comparison between participants with poor and good sleep quality

Compared with participants with good sleep quality, participants with poor sleep quality were older (71.4 ± 3.9 years vs. 69.7 ± 4.4. years, *p* = 0.04) and had a significantly shorter daily sleep duration on weekdays (5.46 ± 1.55 h vs. 7.38 ± 1.18 h, *p* = 0.01) and weekends (5.75 ± 1.61 h vs. 7.50 ± 1.21 h, *p* = 0.01) as well as a higher prevalence of insomnia (97.1 vs. 44.7%, *p* = 0.01) and sleep apnea (38.2 vs. 16.9%, *p* = 0.01). Additionally, compared with participants with good sleep quality, participants with poor sleep quality were more likely to have psychiatric comorbidities (anxiety, 52.9 vs. 11.4%; depression, 70.6 vs. 16.9%; both *p* = 0.01), dyslipidemia (17.6 vs. 3.8%, *p* = 0.01), and sleeping pill intake (23.5 vs. 1.7%, *p* = 0.01). Compared to participants with good sleep quality, those with poor sleep quality had a more frequent EDS and restless legs syndrome, but these differences were not statistically significant. Finally, there were no between-group differences in the frequency of sociodemographic risk factors, including low educational attainment, inoccupation, drinking alcohol, smoking, lack of exercise, obesity, and shift work (Table 2).

**Table 2:** Comparison between elderly individuals with good and poor sleep quality.

**Characteristics**	**Poor sleep (*N* = 34)**	**Good sleep (*N* = 237)**	***p*-value**
Mean age (years)	71.4 ± 3.9	69.7 ± 4.4	0.04
Female	21 (61.8)	118 (49.8)	0.19
**Level of education**			
Under middle school graduate	24 (70.6)	176 (74.3)	0.65
Over high school graduate	10 (29.4)	61 (25.7)	
Inoccupation	11 (32.4)	63 (26.6)	0.48
Alcohol	7 (20.6)	60 (25.3)	0.55
Current smoker	3 (8.8)	36 (15.2)	0.32
Lack of exercise	22 (64.7)	120 (50.6)	0.30
Obesity	9 (26.5)	63 (26.6)	0.99
Hypertension	20 (58.8)	103 (43.5)	0.09
Diabetes mellitus	7 (20.6)	41 (17.3)	0.64
Cardiovascular disease	14 (41.2)	72 (30.4)	0.21
Dyslipidemia	6 (17.6)	9 (3.8)	0.01
Sleep duration during weekdays (hours)	5.46 ± 1.55	7.38 ± 1.18	0.01
Sleep duration during weekends (hours)	5.75 ±1.61	7.50 ± 1.21	0.01
Insomnia	33 (97.1)	106 (44.7)	0.01
Daytime sleepiness	9 (26.5)	53 (22.4)	0.59
Sleep apnea	13 (38.2)	40 (16.9)	0.01
Restless leg syndrome	4 (11.8)	24 (10.1)	0.77
Anxiety	18 (52.9)	27 (11.4)	0.01
Depression	24 (70.6)	40 (16.9)	0.01
Shift work	1 (5.0)	16 (10.0)	0.47
On sleeping pills	8 (23.5)	4 (1.7)	0.01

Data are expressed as mean ± standard deviation or number (percentage).

*p*-value was calculated by χ^2^ test or student's *t*-test; alcohol, refers to drinking more than four times a week; lack of exercise, refers to no exercise at all; obesity, refers to BMI ≥ 25; cardiovascular disease, refers to coronary heart disease, stroke, peripheral arterial disease, aortic disease; poor sleep quality, PSQI score ≥ 9; insomnia, ISI score ≥ 8; daytime sleepiness, ESS score ≥ 10; sleep apnea, BQ score ≥ 2; RLS, meets all four criteria of the CH-RLSq; anxiety, GAS score ≥ 5; depression, PHQ-9 score ≥ 5.

#### 3.3.2. Comparison between patients with and without EDS

There was no significant age difference between patients with and without EDS (69.3 ± 4.0 vs. 70.1 ± 4.4 years, *p* = 0.17). There was a significantly higher proportion of women among participants with EDS than among those without (66.1 vs. 46.9%, *p* = 0.01).

Respondents with EDS were more likely to have insomnia (66.1 vs. 46.9%, *p* = 0.01) and depression (33.9 vs. 20.6%, *p* = 0.03) than those without EDS. More frequent anxiety, poor sleep quality, sleep apnea, restless legs syndrome, and shorter sleep durations on weekdays (6.98 ± 1.52 h vs. 7.19 ± 1.34 h, *p* = 0.33) and weekends (7.11 ± 1.46 h vs. 7.33 ± 1.37 h, *p* = 0.27) were also noted among those with excessive daytime sleepiness, but these differences were not statistically significant.

There was no significant between-group difference in educational attainment, occupational status, alcohol drinking, and shift work; however, participants with EDS had a significantly lower frequency of current smoking than those without (3.2 vs. 17.7%, *p* = 0.01). Finally, there was no significant between-group difference in the use of sleeping pills (Table 3).

**Table 3:** Comparison between elderly patients with and without excessive daytime sleepiness.

**Characteristics**	**With EDS (*N* = 62)**	**Without EDS (*N* = 209)**	***p*-value**
Mean age (years)	69.3 ± 4.0	70.1 ± 4.4	0.17
Female	41 (66.1)	98 (46.9)	0.01
**Level of education**			
Under middle school graduate	47 (75.8)	153 (73.2)	0.68
Over high school graduate	15 (24.2)	56 (26.8)	
Inoccupation	14 (22.6)	60 (28.7)	0.34
Alcohol	15 (24.2)	52 (24.9)	0.91
Current smoker	2 (3.2)	37 (17.7)	0.01
Lack of exercise	39 (62.9)	103 (49.3)	0.15
Obesity	15 (24.2)	57 (27.3)	0.63
Hypertension	22 (35.5)	101 (48.3)	0.07
Diabetes mellitus	16 (25.8)	32 (15.3)	0.06
Cardiovascular disease	24 (38.7)	62 (29.7)	0.18
Dyslipidemia	1 (1.6)	14 (6.7)	0.12
Sleep duration during weekdays (hours)	6.98 ± 1.52	7.19 ± 1.34	0.33
Sleep duration during weekends (hours)	7.11 ± 1.46	7.33 ± 1.37	0.27
Poor sleep quality	9 (14.5)	25 (12.0)	0.59
Insomnia	41 (66.1)	98 (46.9)	0.01
Sleep apnea	16 (25.8)	37 (17.7)	0.16
Restless leg syndrome	7 (11.3)	21 (10.0)	0.78
Anxiety	15(24.2)	30 (14.4)	0.07
Depression	21 (33.9)	43 (20.6)	0.03
Shift work	5 (11.9)	12 (8.7)	0.53
On sleeping pills	2 (3.2)	10 (4.8)	0.60

Data are expressed as mean ± standard deviation or number (percentage).

*p*-value was calculated by χ^2^ test or student's *t*-test; alcohol, refers to drinking more than four times a week; Lack of exercise, refers to no exercise at all; obesity, refers to BMI ≥ 25; cardiovascular disease, refers to coronary heart disease, stroke, peripheral arterial disease, aortic disease; poor sleep quality, PSQI score ≥ 9; insomnia, ISI score ≥ 8; daytime sleepiness, ESS score ≥ 10; sleep apnea, BQ score ≥ 2; RLS, meets all four criteria of the CH-RLSq; anxiety, GAS score ≥ 5; depression, PHQ-9 score ≥ 5.

#### 3.3.3. Comparison between patients with and without insomnia

There was no significant difference in the mean age according to the presence of insomnia (70.2 ± 4.4 vs. 69.7 ± 4.3, *p* = 0.48). Compared with participants without insomnia, those with insomnia showed a higher proportion of women (60.4 vs. 41.7%, *p* = 0.01). Moreover, in the group with insomnia, the rate of lacking exercise was higher (60.4 vs. 43.9%, *p* = 0.02). There was no significant difference in the frequency of hypertension, diabetes mellitus, and dyslipidemia according to the presence of insomnia; however, participants with insomnia had a higher prevalence of cardiovascular disease (38.1 vs. 25.0%, *p* = 0.02). While poor sleep quality, anxiety, and depression were more prevalent in the group with insomnia, there was no significant difference in the prevalence of sociodemographic factors (Table 4).

**Table 4:** Comparison between elderly individuals with and without insomnia.

**Characteristics**	**With insomnia (*N* = 139)**	**Without insomnia (*N* = 132)**	***p*-value**
Mean age (years)	70.2 ± 4.4	69.7 ± 4.3	0.48
Female	84 (60.4)	55 (41.7)	0.01
**Level of education**			
Under middle school graduate	102 (73.4)	98 (74.2)	0.87
Over high school graduate	37 (26.6)	34 (25.8)	
Inoccupation	40 (28.8)	34 (25.8)	0.58
Alcohol	32 (23.0)	35 (26.5)	0.51
Current smoker	19 (13.7)	20 (15.2)	0.73
Lack of exercise	84 (60.4)	58 (43.9)	0.02
Obesity	30 (21.6)	42 (31.8)	0.06
Hypertension	60 (43.2)	63 (47.7)	0.45
Diabetes mellitus	29 (20.9)	19 (14.4)	0.16
Cardiovascular disease	53 (38.1)	33 (25.0)	0.02
Dyslipidemia	9 (6.5)	6 (4.5)	0.49
Sleep duration during weekdays (hours)	6.86 ± 1.41	7.44 ± 1.29	0.64
Sleep duration during weekends (hours)	6.97 ± 1.41	7.61 ± 1.29	0.80
Poor sleep quality	33 (23.7)	1 (0.8)	0.01
Excessive daytime sleepiness	41 (29.5)	21 (15.9)	0.01
Sleep apnea	30 (21.6)	23 (17.4)	0.39
Restless leg syndrome	18 (12.9)	10 (7.6)	0.15
Anxiety	37 (26.6)	8 (6.1)	0.01
Depression	59 (42.4)	5 (3.8)	0.01
Shift work	8 (8.6)	9 (10.3)	0.69
On sleeping pills	12 (8.6)	0 (0.0)	0.01

Data are expressed as mean ± standard deviation or number (percentage).

*p*-value was calculated using the χ^2^ test or Student's *t*-test; alcohol, refers to drinking more than four times a week; lack of exercise, refers to no exercise at all; obesity, refers to BMI ≥ 25; cardiovascular disease, refers to coronary heart disease, stroke, peripheral arterial disease, aortic disease; poor sleep quality, PSQI score ≥ 9; insomnia, ISI score ≥ 8; daytime sleepiness, ESS score ≥ 10; sleep apnea, BQ score ≥ 2; RLS, meets all four criteria of the CH-RLSq; anxiety, GAS score ≥ 5; depression, PHQ-9 score ≥ 5.

### 3.4. Factors affecting sleep quality, daytime sleepiness, and insomnia

#### 3.4.1. Risk factors for poor sleep quality in the elderly population

Multivariate logistic regression analysis revealed that hypertension (OR, 2.51; 95% CI, 1.01–6.26; *p* = 0.05), dyslipidemia (OR, 6.62; 95% CI, 1.56–28.10; *p* = 0.01), insomnia (OR, 29.90; 95% CI, 3.13–286.10; *p* = 0.01), anxiety (OR, 4.15; 95% CI, 1.53–11.28; *p* = 0.01), and depression (OR, 2.93; 95% CI, 1.10–7.83; *p* = 0.03) were independent risk factors for poor sleep quality (Table 5).

**Table 5:** Risk factors for poor sleep quality, excessive daytime sleepiness, and insomnia in the elderly population.

**Factors**	**Poor sleep quality**			**Excessive daytime sleepiness**			**Insomnia**		

	**OR**	**95% CI**	***p*** **-value**	**OR**	**95% CI**	***p*** **-value**	**OR**	**95% CI**	***p*** **-value**
Age	1.09	0.99, 1.21	0.09	0.94	0.87, 1.01	0.09	1	0.93, 1.07	0.99
Female	0.98	0.30, 3.14	0.97	2.03	1.09, 3.78	0.03	1.62	0.92, 2.87	0.1
Hypertension	2.51	1.01, 6.26	0.05	0.41	0.19, 0.87	0.02	0.91	0.49, 1.68	0.76
Diabetes mellitus	0.63	0.20, 2.00	0.43	1.86	0.89, 3.88	0.1	1.14	0.51, 2.54	0.75
Cardiovascular disease	1.8	0.67, 4.85	0.25	1.41	0.72, 2.75	0.31	1.83	0.99, 3.38	0.05
Dyslipidemia	6.62	1.56, 28.10	0.01	0.19	0.02, 1.50	0.12	0.6	0.13, 2.71	0.51
Poor sleep quality				0.86	0.30, 2.42	0.77	19.73	2.49, 156.67	0.01
Excessive daytime sleepiness	0.98	0.32, 2.98	0.97				1.82	0.91, 3.63	0.09
Insomnia	29.9	3.13, 286.10	0.01	1.92	1.03, 3.59	0.04			
Restless leg syndrome	0.57	0.14, 2.44	0.45	1.17	0.44, 3.14	0.76	1.46	0.57, 3.77	0.43
Sleep apnea	1.75	0.61, 5.06	0.3	2.84	1.20, 6.73	0.02	0.96	0.40, 2.32	0.94
Anxiety	4.15	1.53, 11.28	0.01	1.43	0.65, 3.16	0.38	1.54	0.56, 4.25	0.41
Depression	2.93	1.10, 7.83	0.03	1.19	0.52, 2.72	0.68	11.48	4.24, 31.06	0.01
Inoccupation	1.48	0.53, 4.14	0.46	0.95	0.43, 2.11	0.91	1.39	0.70, 2.78	0.35
Low level of education	0.52	0.18, 1.45	0.21	1.11	0.52, 2.39	0.79	0.81	0.41, 1.62	0.55

Cardiovascular disease refers to coronary heart disease, stroke, peripheral arterial disease, and/or aortic disease; poor sleep quality, PSQI score ≥ 9; Insomnia, ISI score ≥ 8; daytime sleepiness, ESS score ≥ 10; sleep apnea, BQ score ≥ 2; RLS, meets all four criteria of the CH-RLSq; anxiety, GAS score ≥ 5; depression, PHQ-9 score ≥ 5; low educational attainment, refers to under middle school graduate; OR, odd's ratio; CI, confidence interval.

#### 3.4.2. Risk factors for EDS in the elderly population

Multivariate logistic regression analysis revealed that female sex (OR, 2.03; 95% CI, 1.09–3.78; *p* = 0.03), insomnia (OR, 2.84; 95% CI, 1.03–3.59; *p* = 0.04), and sleep apnea (OR, 2.84; 95% CI, 1.20–6.73; *p* = 0.02) were independent risk factors for EDS; in contrast, the presence of hypertension decreased the risk of EDS (OR, 0.41; 95% CI, 0.19–0.87; *p* = 0.02) (Table 5).

#### 3.4.3. Risk factors for insomnia in the elderly population

Multivariate logistic regression analysis revealed that poor sleep quality (OR, 19.73; 95% CI, 2.49–156.67) and depression (OR, 11.48; 95% CI, 4.24–31.06) were independent risk factors for insomnia (Table 5).

## 4. Discussion

In our study, 12.5, 22.9, and 51.3% of the participants had poor sleep quality, EDS, and insomnia, respectively. Further, sleep status in the elderly population was influenced by various factors, including age, sex, and metabolic and psychiatric comorbid diseases.

Comparing sleep indices including PSQI, ISI, and ESS, it was found that female had worse sleeping habits even in the elderly population. Female have shown higher prevalence of insomnia compared to male according to previous studies (46–48). Similar results were also found in this study in the elderly population. According to a study of young adult population, female showed higher prevalence of poor sleep quality, even after adjusting psychiatric and sociodemographic factors (49).

The frequency of arousal during sleep increases with age, which causes difficulty in maintaining sleep, reduced sleep duration and decreased slow-wave sleep (50). Over 50% of individuals aged > 65 years have at least one sleep problem, including trouble falling asleep, waking up, waking up too early, needing to nap, and not feeling rested (51). Since sleep patterns vary according to health status and environment, there are wide variations in the clinical characteristics of sleep disorders in the elderly population (31). Our study performed an overall review of the sleep status in the general, rather than only healthy, elderly population.

The total sleep duration decreases with age (50). The SIESTA database showed that the total sleep time in the adult population decreased by ~8–10 min per 10 years in the adult population; however, physiological sleep changes usually stop at the age of ~60 years (52). Since even older adults without complaints of sleep problems present with significant sleep disturbance upon objective evaluation, it is necessary to properly evaluate sleep quality in the elderly population (53). A survey study on 1,085 individuals aged ≥ 75 years reported poor sleep quality in 16.6% of the participants (54), which is consistent with our finding that 34 (12.5%) participants had poor sleep quality.

Participants with poor sleep quality had shorter sleep duration on both weekdays and weekends, with this difference being higher than that previously reported (53). Among the sleep problems, insomnia is a predictor of poor sleep quality. Specifically, since patients with insomnia have a greater need for good sleep quality, they are more vulnerable to poor sleep quality (55).

We found that underlying metabolic comorbidities, including hypertension and dyslipidemia, as well as psychiatric comorbidities, including anxiety and depression, increased the risk of poor sleep quality in the elderly population. Subjective sleep problems in the elderly population are influenced by comorbidities. Our findings are consistent with previous reports (53). Several environmental factors have been reported to affect sleep status (11, 14); however, we observed no correlation between occupational status and educational attainment with the presence of poor sleep quality.

In our study, 62 (22.9%) participants reported EDS, which is consistent with a previously reported prevalence of 20% among adults aged 65 years (56). The female sex was predominant among participants with EDS and was an independent predictor of EDS in the elderly Korean population. Contrastingly, a Swiss study on individuals aged 35–75 years reported that the male sex was a predictor of EDS (57). However, consistent with this previous study, the presence of insomnia and sleep apnea could predict excessive daytime (57).

Hypertension was a negative predictor of EDS in the elderly population. A previous study reported that total ESS scores decreased with age in hypertensive patients with sleep apnea (58). Furthermore, a 6-year follow-up study reported that EDS was a risk factor for mortality related to cardiovascular diseases, including hypertension, in the elderly population (59). This suggests an inter-relationship between EDS and hypertension; however, further studies are warranted.

EDS worsens physical and functional activity in the elderly population (60). A French study reported that EDS negatively affected cognitive function in the elderly population and that a high EDS score could be an early modifiable risk factor (61). Therefore, it is important to evaluate EDS in the elderly population.

The prevalence of insomnia in our study was 51.3%, which was higher than that in a previous Korean study on adults aged 20–69 years (32). Since previous studies have reported a prevalence of insomnia of 12–20% in the elderly population (62), our reported prevalence was rather high. This could be attributed to our definition of subthreshold insomnia as an ISI score ≥ 8. However, another study has reported that ~ 50% of the older adult population presents with insomnia symptoms, including difficulty initiating or maintaining sleep (63).

In our study, poor sleep quality and depression were independent predictors of insomnia in the elderly population. It is well-known that clinicians should consider psychiatric comorbidities when treating insomnia. Our study shares the same clinical context (64). The correlation between sleep problems and psychiatric problems has been studied in the past (65), and more active research is needed in the area of appropriate treatment in the future.

This study has several limitations. First, we used questionnaires administered by interviewers; it is difficult to determine whether the elderly respondents comprehended the question well and provided proper responses since we did not conduct a baseline assessment of their cognitive function status. Furthermore, questionnaire findings cannot accurately reflect objective sleep quality (66). Accordingly, it is difficult to measure sleep status and quality in the elderly population (66, 67).

Second, chronic diseases including neurological disorders such as history of stroke, neurodegenerative disease, head trauma, primary and secondary headache in the elderly population have been scantily studied. About 93% of the elderly population presents with chronic diseases (68). In the future, when planning a study on sleep in the elderly population, the evaluation of chronic diseases should be thoroughly considered since sleep problems have various causes.

Third, although this was a nationwide study, our sample size was small due to our low cooperation rate (36.2%) (69). Therefore, future large-scale studies are warranted to reliably establish the prevalence and predictors of sleep disorders in the elderly population.

Lastly, since this is a study using data prior to COVID-19, which had a global impact, there are limitations in directly incorporating it into the sleep of the current elderly population. Additional research is needed on the relationship between COVID-19 infection and sleep status in the elderly.

## 5. Conclusions

A substantial proportion of elderly individuals experience poor sleep quality, EDS, and insomnia. Factors influencing sleep problems included age, sex, and metabolic and psychiatric comorbidities. It is important to determine the sleep status in the elderly population since it affects overall health, including cognitive and metabolic function.

## Data availability statement

The raw data supporting the conclusions of this article will be made available by the authors, without undue reservation.

## Ethics statement

The study protocol was approved by the Institutional Review Board/Ethics Committee of Hallym University Sacred Heart Hospital in Korea (Approval no. 2011-I077). The patients/participants provided their written informed consent to participate in this study.

## Author contributions

HH designed the study, analyzed the data, and wrote the article. KK supervised data analysis. C-HY, KY, and MC collected data. W-JK designed the study, analyzed the data, and assisted in writing the article. All authors have reviewed and approved the final version of the manuscript.
