# Supplementary material for: Sleep state of the elderly population in Korea: Nationwide cross-sectional population-based study
Source: Front Neurol. 2023 Jan 9;13:1095404. doi: 10.3389/fneur.2022.1095404 (PMC9868806; doi:10.3389/fneur.2022.1095404)
Supplement: Supplementary file 1 [file Table_1.docx]

| Supplementary table 1. Comparison of sleep related scales between elderly individuals with different gender. | | | |
| --- | --- | --- | --- |
| Characteristics | Female (N=139) | Male (N=132) | p-value |
| Mean age (years) | 70.21 ± 4.44 | 69.66 ± 4.26 | 0.30 |
| Sleep duration during weekdays (hours) | 7.14 ± 1.38 | 7.14 ± 1.39 | 0.98 |
| Sleep duration during weekends (hours) | 7.25 ± 1.32 | 7.31 ± 1.46 | 0.72 |
| PSQI | 5.63 ± 2.71 | 4.80 ± 2.83 | 0.01 |
| ISI | 10.90 ± 6.75 | 8.95 ± 6.69 | 0.02 |
| ESS | 6.91 ± 4.30 | 5.71 ± 4.15 | 0.02 |
| PHQ-9 | 3.48 ± 3.98 | 2.29 ± 3.66 | 0.01 |
| GAS | 2.53 ± 2.28 | 1.84 ± 2.29 | 0.01 |
| Data are expressed as mean ± standard deviation. | | | |
| p-value was calculated using the χ2 test; PSQI, refers to Pittsburgh Sleep Quality Index; ISI, refers to Insomnia Severity Index; ESS, refers to Epworth Sleepiness Scale; PHQ-9, refers to Patient Health Qeustionnaire-9; GAS, refers to Goldberg Anxiety Scale. | | | |
